# Administration of bifidobacterium and lactobacillus strains modulates experimental myasthenia gravis and experimental encephalomyelitis in Lewis rats

**DOI:** 10.18632/oncotarget.25170

**Published:** 2018-04-27

**Authors:** Alessandra Consonni, Chiara Cordiglieri, Elena Rinaldi, Roberta Marolda, Ilaria Ravanelli, Elena Guidesi, Marina Elli, Renato Mantegazza, Fulvio Baggi

**Affiliations:** ^1^ Neuroimmunology and Neuromuscular Diseases Unit, Neurological Institute ‘Carlo Besta’, Milan, Italy; ^2^ AAT-Advanced Analytical Technologies, Fiorenzuola d’Arda, Piacenza, Italy

**Keywords:** probiotics, experimental autoimmune myasthenia gravis, experimental autoimmune encephalomyelitis, Immunology

## Abstract

Probiotics beneficial effects on the host are associated with regulation of the intestinal microbial homeostasis and with modulation of inflammatory immune responses in the gut and in periphery. In this study, we investigated the clinical efficacy of two lactobacillus and two bifidobacterium probiotic strains in experimental autoimmune myasthenia gravis (EAMG) and experimental autoimmune encephalomyelitis (EAE) models, induced in Lewis rats. Treatment with probiotics led to less severe disease manifestation in both models; *ex vivo* analyses showed preservation of neuromuscular junction in EAMG and myelin content in EAE spinal cord. Immunoregulatory transcripts were found differentially expressed in gut associated lymphoid tissue and in peripheral immunocompetent organs. Feeding EAMG animals with probiotics resulted in increased levels of Transforming Growth Factor-β (TGFβ) in serum, and increased percentages of regulatory T cells (Treg) in peripheral blood leukocyte. Exposure of immature dendritic cells to probiotics induced their maturation toward an immunomodulatory phenotype, and secretion of TGFβ. Our data showed that bifidobacteria and lactobacilli treatment effectively modulates disease symptoms in EAMG and EAE models, and support further investigations to evaluate their use in autoimmune diseases.

## INTRODUCTION

In the recent years, there has been increasing interest on the role of the intestinal microbiota in health and disease, as well as on the use of probiotics to modulate its activity [1–3]. The gastro intestinal tract (GIT) and the central nervous system (CNS) are connected through a network of neuroendocrine and immunological signaling pathways, collectively referred as the gut–brain axis [4–6]. Nutritional and energetic levels are monitored thanks to the gut microbiota, which conveys information from the ingested aliments (i.e., vitamins, minerals, carbohydrates, fats, etc.) to the CNS via the gut–brain axis; specific neurological pathways have evolved to respond to microbial commensals of the gut, either directly via microbial metabolites or indirectly by the immune, metabolic, or endocrine systems. Bacterial colonization of the gut influences the development and functions of the immune system; indeed, an impaired intestinal barrier might lead to an imbalanced Thelper Th1/Th2 response, thus triggering gastrointestinal autoimmune diseases [7, 8]. Probiotics are defined as a “live microorganisms that, when administered in adequate amounts, confer a health benefit on the host” [9]. Commensal bacteria in the gut are often the source of probiotic strains, and two common general benefits are associated with probiotics: supporting a healthy digestive tract and a healthy immune system [9]. Probiotic treatment can promote the induction or restoration of regulatory-type immune responses [10–12], by modulating the balance between pro- and anti-inflammatory cytokines [13–15], enhancing the generation of IL10^+^, TGFβ^+^ and COX2^+^ regulatory DCs [16], and enriching CD4^+^CD25^+^ regulatory T cell (Treg) cells [17, 18].

Hence, the immunomodulatory properties of specific probiotic strains may represent a promising therapeutic strategy for allergic and chronic inflammatory diseases [3, 10, 19, 20]. Among the human microbiota, lactobacilli, mainly predominant in the gastric region and upper gastro-intestinal tract, and bifidobacteria, present in the lower intestinal tract, showed therapeutic effects either in experimentally induced colitis [21], experimental inflammatory bowel disease [22], experimental arthritis [23], and in infants with atopic dermatitis [24, 25].

Myasthenia gravis (MG) and multiple sclerosis (MS) are the most common neurological autoimmune diseases [26, 27], with a high impact on everyday life; long-term drug treatments are required for most patients and must be individually tailored in order to minimize adverse effects. MG is an autoimmune disease of the neuromuscular junction (NMJ), leading to muscle weakness and fatigability; the nicotinic acetylcholine receptor (AChR) is the main auto-antigen recognized by pathogenic antibodies in more than 75–80% of patients, although other molecular targets are recognized [26]. Experimental autoimmune myasthenia gravis (EAMG) in the susceptible Lewis rat is a well-established model useful to elucidate the pathogenic mechanism of the disease and to develop new or improved MG therapies [28, 29]. MS is a chronic inflammatory, T cell-dependent, autoimmune disease of the central nervous system (CNS), in which the interplay between inflammatory and neurodegenerative aspects results in neurological relapses followed by progressive accumulation of disability [30]. The experimental autoimmune encephalomyelitis (EAE) animal models share several clinical, pathogenic and immunological features with the human disease, and can be used as prototype-model for new therapies [31–34]. Lewis rat is widely used for EAE studies, due to its susceptibility to disease induction with relatively low doses of guinea pig MBP in CFA with remarkable reproducibility, without additional adjuvants (e.g., pertussis toxin used for EAE induction in mice) [35]. The monophasic EAE model is characterized by severe hind limb paralysis at day 18–20 after immunization [36] followed by a recovery phase characterized by increased Th17 and Treg cells [37].

In the present study, we investigated the immunomodulatory effect of two Lactobacillus strains (LMG P-23257 and ATCC 53103) and two Bifidobacterium strains (BB12^®^, and LMG S-28195) in rat EAMG and EAE models. The selected bacteria were known to produce anti-oxidant and anti-inflammatory molecules, such as conjugated linoleic acid (CLA) [21, 38, 39]. We observed amelioration of clinical symptoms in both experimental models. *Ex vivo* analyses demonstrated differential expression of inflammatory and regulatory markers in gut lymphoid tissue (Peyer's patches, PPs), leading to modulation of the autoimmune attack to the target structures, the NMJ in EAMG and the spinal cord in EAE. *In vitro* experiments demonstrated that maturation of dendritic cells (DCs) exposed to probiotics resulted in increased TGFβ expression and release; indeed, TGFβ differential expression was found in the gut lymphoid tissue as well as in the thymus of EAMG animals and in the spinal cord of EAE animals. Our data demonstrate that our Bifidobacterium and Lactobacillus strains are effective in inducing immunological tolerance to AChR and myelin basic protein (MBP), the target antigens in MG and MS; further studies need to be performed to evaluate the use of probiotics as a potential therapeutic approach for these autoimmune diseases.

## RESULTS

### Lactobacilli and bifidobacteria ameliorate experimental autoimmune myasthenia gravis

**Figure 1:**
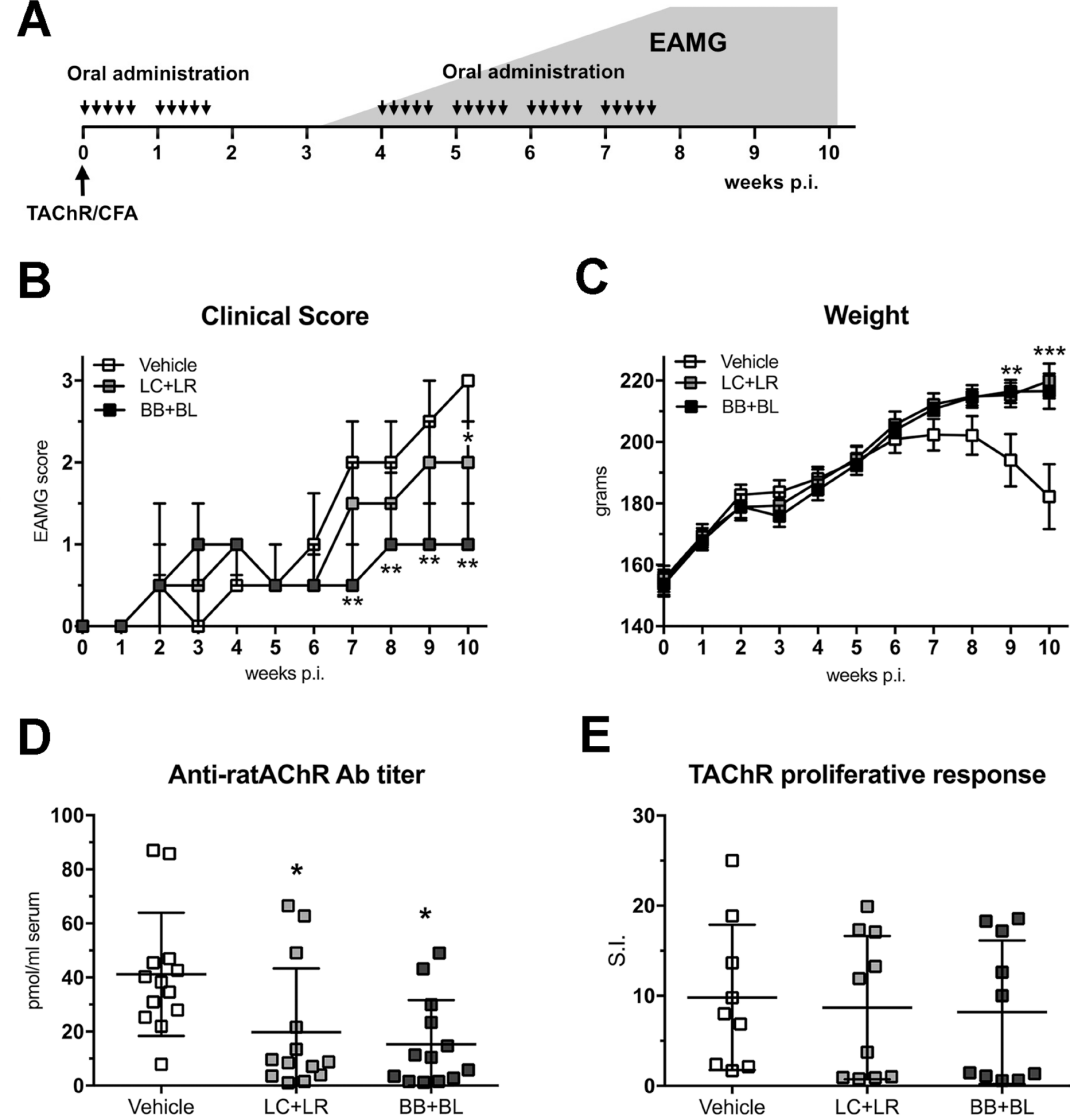
Lactobacillus and bifidobacterium probiotic strains led to EAMG amelioration (**A**) Experimental design: EAMG induction and probiotic treatments (30 administrations, 2 × 10^9^ CFU, 300 μl PBS). The grey area represents typical EAMG course in untreated animals. (**B**) Clinical EAMG score (median and IQR) and (**C**) body weight (mean ± SEM) of EAMG animals treated with vehicle (*n* = 14), LC+LR (*n* = 15), and BB+BL (*n* = 15). (**D**) Anti-ratAChR Ab serum titers (pmol/ml of rat serum, mean ± SD) and (**E**) LNCs responses to TAChR (stimulation index, S.I., mean ± SD) in EAMG animals treated with vehicle, LC+LR, and BB+BL. Statistical significance was assessed by Kruskal-Wallis test; *p*-values were corrected for multiple-comparisons. One-way ANOVA test with Dunnett's multiple comparison test was used for body weight, anti-ratAChR and LNC responses analyses. Scatter dot plots with mean ± SD. ^*^P < 0.05, ^**^P < 0.01, ^***^P < 0.001.

*Torpedo californica* AChR (TAChR)-immunized Lewis rats were treated with combinations of lactobacilli (LC+LR) or bifidobacteria (BB+BL), (Figure 1A); probiotic treatments were given in correspondence of the acute IgM-mediated and the chronic IgG-mediated phases, characteristic of the rat EAMG model. LC+LR and BB+BL administration ameliorated EAMG manifestations (clinical score, Figure 1B; body weight, Figure 1C). A significant reduction in EAMG score was observed during the treatment with BB+BL, starting from week 7 p.i., (Figure 1B); also LC+LR treatment was able to significantly ameliorate EAMG, but from week 9 p.i. (Figure 1B). EAMG clinical score at the end of experiment (week 10) were: BB+BL group median score = 1 (IQR1–1.5, *P* < 0.01 compared to vehicle); LC+LR group median score = 2 (IQR 1.5–2.5, *P* < 0.05 compared to vehicle) and vehicle group median score = 3 (IQR 2–3).

Probiotic-treated EAMG animals showed a normal growth rate compared to untreated EAMG (Figure 1C). EAMG improvement was associated with a significant decrease of anti-ratAChR antibody serum titers (LC+LR group: 19.8 pmol/ml ± 23.5 SD, *P* < 0.05; BB+BL group: 18.9 pmol/ml ± 20.9 SD, *P* < 0.05; vehicle-fed EAMG rats: 41.2 pmol/ml ± 22.7 SD; Figure 1D). TAChR proliferative responses (expressed as stimulation index) from draining lymph node cells (LNCs) were found not modified in LC+LR and in BB+BL treated EAMG rats compared to vehicle-fed group (Figure 1E).

Probiotic dosage and schedule of administration were preliminarily studied in healthy Lewis rats (Supplementary Figure 1). Lactobacilli were found increased just after one week, and remained stable during the subsequent two weeks; a modest reduction was seen after the wash-out period (Supplementary Figure 1A, left panel). Bifidobacteria progressively increased during the feeding period, and rapidly returned to the baseline level after the wash-out week (Supplementary Figure 1A, right panel). Gut colonization was also assessed by confocal microscopy, revealing the presence of fluorescently-labeled bacteria in proximity to intestinal villi and PPs from naïve Lewis rats fed with a single dose of labeled LR (1 × 10^9^ CFU, single strain) (Supplementary Figure 1B, representative images).

**Figure 2:**
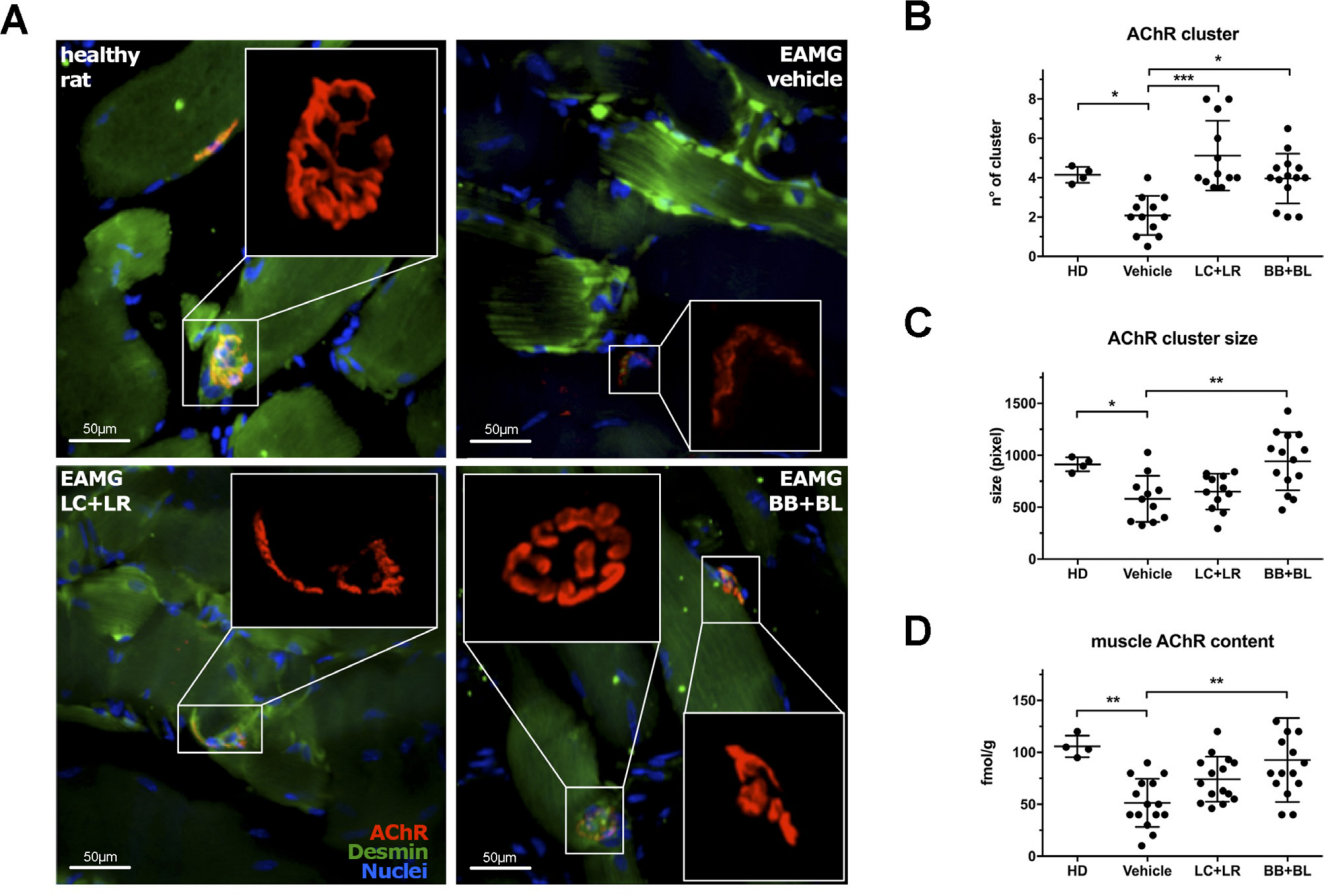
Probiotic-treated EAMG animals showed increased muscle AChR content (**A**) Fluorescence analysis of AChR (αBTX-tetramethylrhodamine, red) in gastrocnemius muscle (Desmin, green) from healthy animal and EAMG rats treated with vehicle, LC+LR, and BB+BL. Confocal microscopy, scale bar 50 μm. Insets show high resolution images of clustered AChR, acquired with 3D-SIM super-resolution microscopy. (**B**) AChR cluster number, (**C**) AChR cluster size (pixel) quantification in gastrocnemius muscles and (**D**) radioimmunoassay determination of total muscle AChR content (fmol/g of tissue) in healthy donor (HD, *n* = 4) and in EAMG rats treated with vehicle, LC+LR or BB+BL (*n* = 11–14). One-way ANOVA test with Dunnett's multiple comparison test was used to calculate statistical significance. Scatter dot plots with mean ± SD ^*^P < 0.05, ^**^P < 0.01, ^***^P < 0.001.

To confirm the clinical improvement in probiotic-treated EAMG animals, we performed a morphological study to detect AChR clusters in the gastrocnemius muscle from healthy rats and EAMG animals treated with vehicle, LC+LR, and BB+BL, by confocal microscopy (Figure 2A) and by super-resolution structured illumination microscopy (Figure 2A, insets). Digitalized images were evaluated for the number (Figure 2B) and for the size (Figure 2C) of AChR clusters. The mean number of AChR clusters was found significantly increased in probiotic-treated animals (LC+LR: 5.1 clusters/image field ± 1.7 SD, *P* < 0.001; BB+BL: 3.9 clusters/image field ± 1.2 SD, *P* < 0.05) compared to vehicle-fed EAMG rats (2.1 clusters/image field ± 0.9 SD); in naïve, age matched, Lewis rats (HD) the number of AChR clusters is 4.2 clusters/image field ± 0.4 SD. The mean AChR cluster size was also found increased in probiotic-treated EAMG rats (LC+LR: 649 pixel ± 172 SD; BB+BL: 942 pixel ± 279 SD, *P* < 0.01), compared to vehicle-treated EAMG (580 ± 222 SD); mean AChR cluster size in normal Lewis rats was 912 pixel ± 67 SD. Muscle AChR content was evaluated by quantitative RIA (Figure 2D): in line with the observed reduction of the anti-ratAChR antibody titers (Figure 1D), probiotic-treated animals showed increased muscle AChR content (LC+LR: 74.1 fmol/g ± 21.6 SD; BB+BL: 92.6 fmol/g ± 40.4 SD, *P* < 0.01) compared to vehicle (51.3 fmol/g ± 23.2 SD); AChR content in healthy animals was 105.8 fmol/g ± 10.4 SD.

### Lactobacilli and bifidobacteria ameliorate experimental autoimmune encephalomyelitis

**Figure 3:**
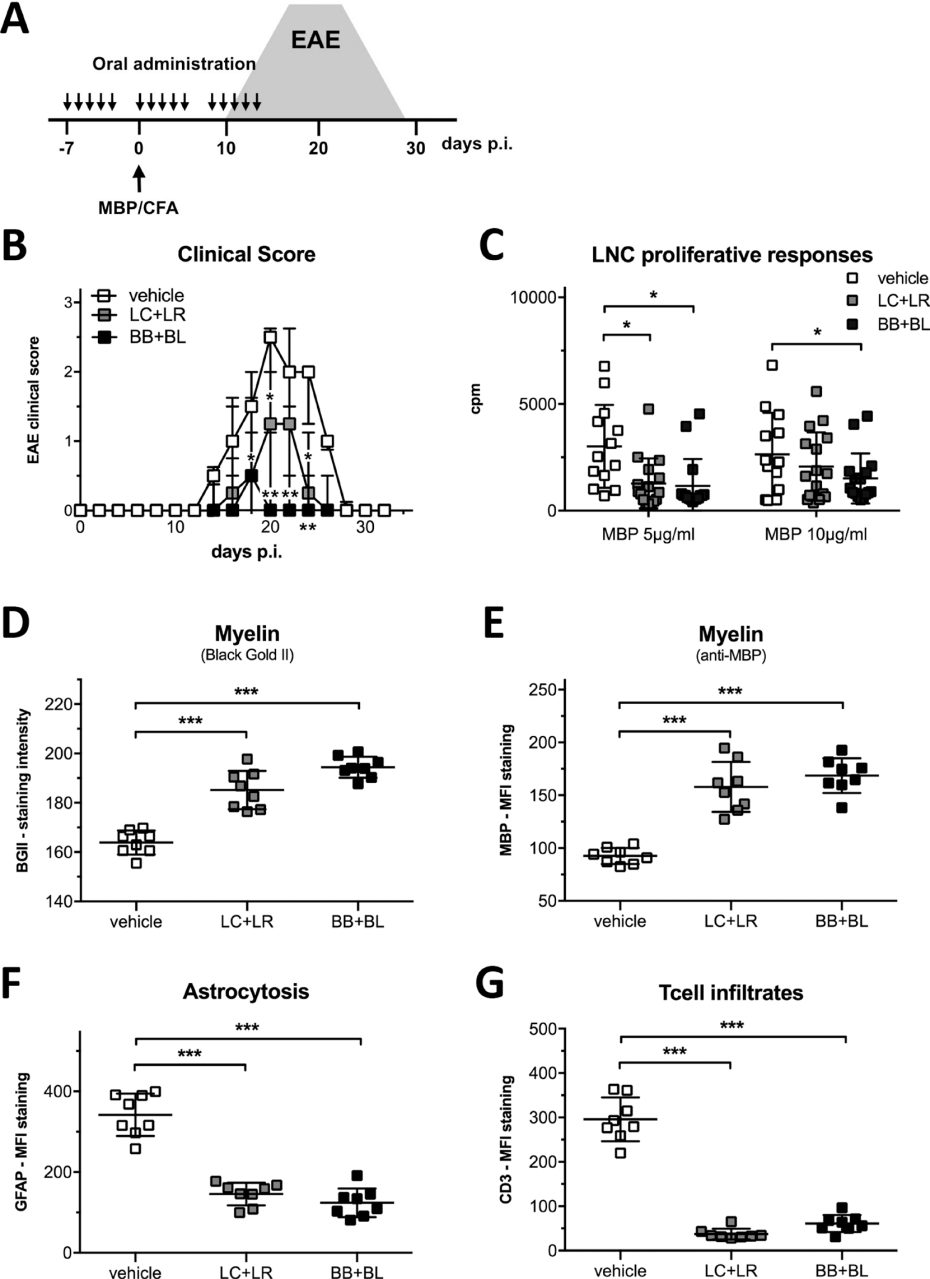
Combinations of lactobacillus and bifidobacterium strains reduced EAE severity (**A**) Experimental design: EAE induction and probiotic treatments (15 administrations, 2 × 10^9^ CFU, 300 μl PBS). The grey area represents typical EAE course in untreated animals. (**B**) Clinical score of EAE animals treated with vehicle, LC+LR, and BB+BL probiotic combination (*n* = 18 rats/group). Data are shown as median and IQR, statistical significance was assessed by Kruskal-Wallis test; *p*-values were corrected for multiple-comparisons. (**C**) *Ex vivo* LNCs proliferative responses to MBP (5 and 10 μg/ml) from EAE animals treated with vehicle, LC+LR, or BB+BL. Background values were ≤147 cpm. Scatter dot plot, mean ± SD. (**D**) Quantifications of myelin content by BGII staining and (**E**) by anti-MBP mAb staining of thoracic spinal cord from EAE rats at disease peak (d = 20) treated with vehicle, LC+LR or BB+BL probiotic combinations (*n* = 8 rats/group). (**F**) Quantifications of reactive astrocytes (anti-GFAP mAb staining) and (**G**) quantification of infiltrating Tcells (anti-CD3 mAb staining) in EAE rats sacrificed at disease peak (d = 20). Representative stainings are reported in Supplementary Figure 3. Statistical significance was assessed by one-way ANOVA test with Dunnett's multiple comparison test. Scatter dot plots with mean ± SD. ^*^P < 0.05, ^**^P < 0.01, ^***^P < 0.001.

Protocol of probiotic administrations in MBP-EAE rat is schematically represented in Figure 3A. EAE clinical manifestations were significantly ameliorated by LC+LR treatment (median score at disease peak: 1.25, IQR 0–2, *P* < 0.05) and by BB+BL treatment (median score at disease peak: 0, IQR 0–1.13, *P* < 0.01) compared to vehicle-fed animals (median score at disease peak: 2.5, IQR 2–2.63) (Figure 3B). In LC+LR and BB+BL treated animals proliferative responses to MBP (5 μg/ml) were significantly reduced (Figure 3C). LNC proliferative responses at MBP 10 μg/ml were reduced only by BB+BL.

Administration of probiotic strains LC, LR, BB and BL was evaluated in the MBP-EAE model, in parallel to the administration of their combinations (LC+LR and BB+BL) (Supplementary Figure 2). Bifidobacteria BB and BL strains did not delay EAE onset, compared to vehicle-fed EAE rats, and a modest reduction in disease severity was observed. BB+BL combination ameliorated EAE manifestation (Supplementary Figure 2A). Treatment with LC and LR strains slightly delayed EAE onset (d = 15 for LR; d = 18 for LC, Supplementary Figure 2B) compared to vehicle-fed EAE (d = 13). Treatment with LC+LR had a similar effect on EAE course to that observed with the single strains. EAE incidence, onset, median score at disease peak (d = 20) and total EAE score are summarized in the Supplementary Figure 2.

Histological and confocal microscopy studies in the spinal cord of EAE rats were focused to detect alteration in myelin sheet, inflammation processes, and presence of infiltrating mononuclear cells, all typical features of the MBP-EAE model. Probiotic treatments (LC+LR and BB+BL) prevented myelin loss in the spinal cord, assessed by quantitative analysis of myelin staining with Black Gold II (Figure 3D) and with anti-MBP mAb (Figure 3E); reduced myelin loss was paralleled with a significant reduction in astrocytosis (GFAP MFI, Figure 3F) and in immune cell infiltration (CD3 MFI, Figure 3G), both markers of an ongoing inflammatory process. Representative spinal cord images for each staining are reported in Supplementary Figure 3 for H/E (panel A), Black Gold II (panel B), MBP- and β Tubulin-immunofluorescence (panel C), GFAP- and CD3-immunofluorescence (panel D).

### Probiotic treatment reduces MBP specific T cell infiltration in EAE spinal cord

**Figure 4:**
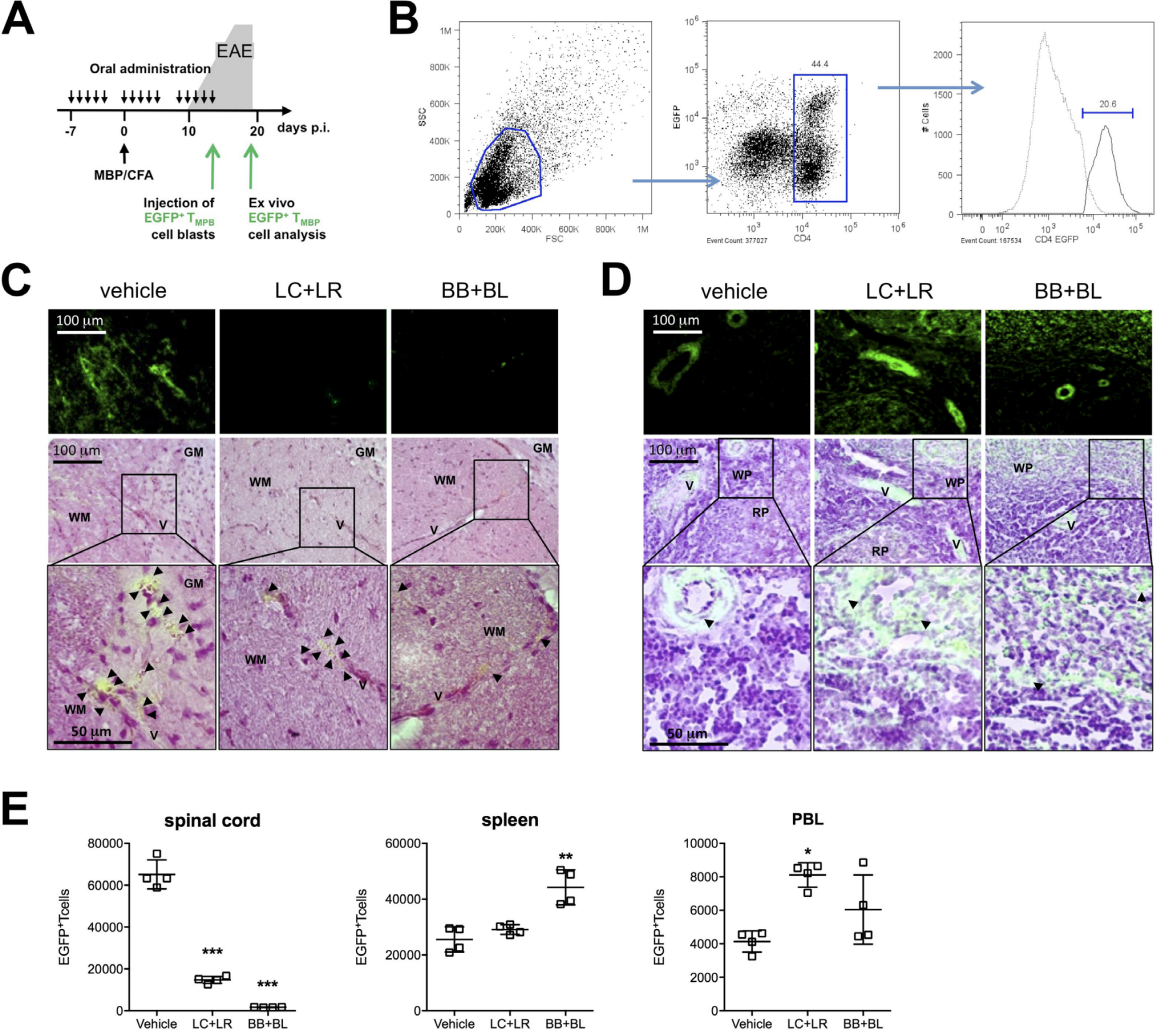
Probiotic treatment prevented immune cell infiltration in the CNS of EAE animals (**A**) Experimental design: EGFP+/–MBP-specific Tcell blasts were injected at day 12–14 in EAE rats; animals were sacrificed 4 days later to assess Tcell blasts infiltration. (**B**) Cytofluorimetric analysis of EGFP+/–MBP-specific Tcell blasts at day 2 post MBP stimulation. (**C**) Thoracic spinal cord and (**D**) spleen EGFP fluorescence images (top - bar scale 100μm) merged with hematoxylin/eosin histological images (middle - bar scale 100 μm; bottom enlarged detail - bar scale 50 μm) to detect infiltrated EGFP+/–MBP-specific Tcell in animals treated with vehicle, LC+LR, and BB+BL. WM: white matter; GM: grey matter; V: vessel; WP: white pulp; RP: red pulp. Black arrow-heads point to EGFP+/–MBP-specific Tcells. (**E**) Quantification of EGFP+/– MBP-specific T cell blasts in lumbar tract of spinal cord, spleen, and peripheral blood (PBL) from EAE rats treated with vehicle, LC+LR, and BB+BL by cytofluorimetric analysis. Data are expressed as number of CD4+EGFP+ T cells in 6 × 10^6^ events for spinal cord, 30 × 10^6^ events for spleen, 3 × 10^6^ events for PBL according to FSC/SSC and CD4/EGFP gates (see Figure 4B). One-way ANOVA test with Dunnett's multiple comparison test. Scatter dot plots with mean ± SD. *N* = 4 rats/group. ^*^P < 0.05, ^**^P < 0.01, ^***^P < 0.001.

To investigate the possible mechanisms associated to the probiotic efficacy in EAE model, EGFP+/– MBP-specific Tcell blasts were i.v. injected in MBP-EAE rats at disease onset (d = 14); rats were fed with vehicle or probiotic combinations (Figure 4A). Tcell blasts were characterized for CD4 and EGFP levels of expression by cytofluorimetric analysis and double positive cells ranged between 18–22% (Figure 4B). By bright field and fluorescence microscopy, we evaluated the presence of exogenous EGFP+/– Tcell blasts in the spinal cord, the CNS site of encephalitogenic T cell infiltration in MBP-EAE model (Figure 4C); the presence of exogenous EGFP+/– Tcells were also evaluated in the spleen (Figure 4D). Four days after i.v. injection, infiltrating EGFP+/– MBP-specific Tcells were quantified in spinal cord single cell suspensions by flow cytometry (Figure 4E); high number of infiltrating EGFP+/– Tcells were found in the spinal cord of vehicle-fed EAE rats, while in bifidobacteria- and lactobacilli-treated animals infiltrated EGFP+/– Tcells were significantly reduced (*p* < 0.001). Quantification of EGFP+/– Tcells in spleen and PBL showed that EGFP+/– Tcells accumulated in peripheral organs of probiotic-fed animals.

### Lactobacilli and bifidobacteria modulate *in vivo* immune-related transcripts in the gut-associated lymphoid tissue and in peripheral organs

PPs were isolated from LC+LR or BB+BL fed naïve rats after 10 doses (T = 2w), or after 15 doses followed by one week washout (T = 4w); by quantitative real time-PCR (qRT-PCR) we analyzed differential expression of FoxP3, TGFβ, CTLA4, and CCR7 mRNAs, compared to vehicle-fed naïve rats. LC+LR but not BB+BL treatment induced upregulation of these transcripts already at T = 2 (Supplementary Figure 1C); on the contrary, the effect of BB+BL was only seen at a later time (T = 4w) (Supplementary Figure 1D), even if probiotic treatment was suspended one week before. The increased expression of FoxP3 mRNA in probiotic fed rats was associated to an increased frequency of CD4^+^CD25^bright^ and FoxP3^+^CD4^+^CD25^bright^ (regulatory) T cells subsets in PPs, mesenteric LNs, and in PBL, with a more robust effect for BB+BL compared to LC+LR (Supplementary Figure 1E–1G).

**Figure 5:**
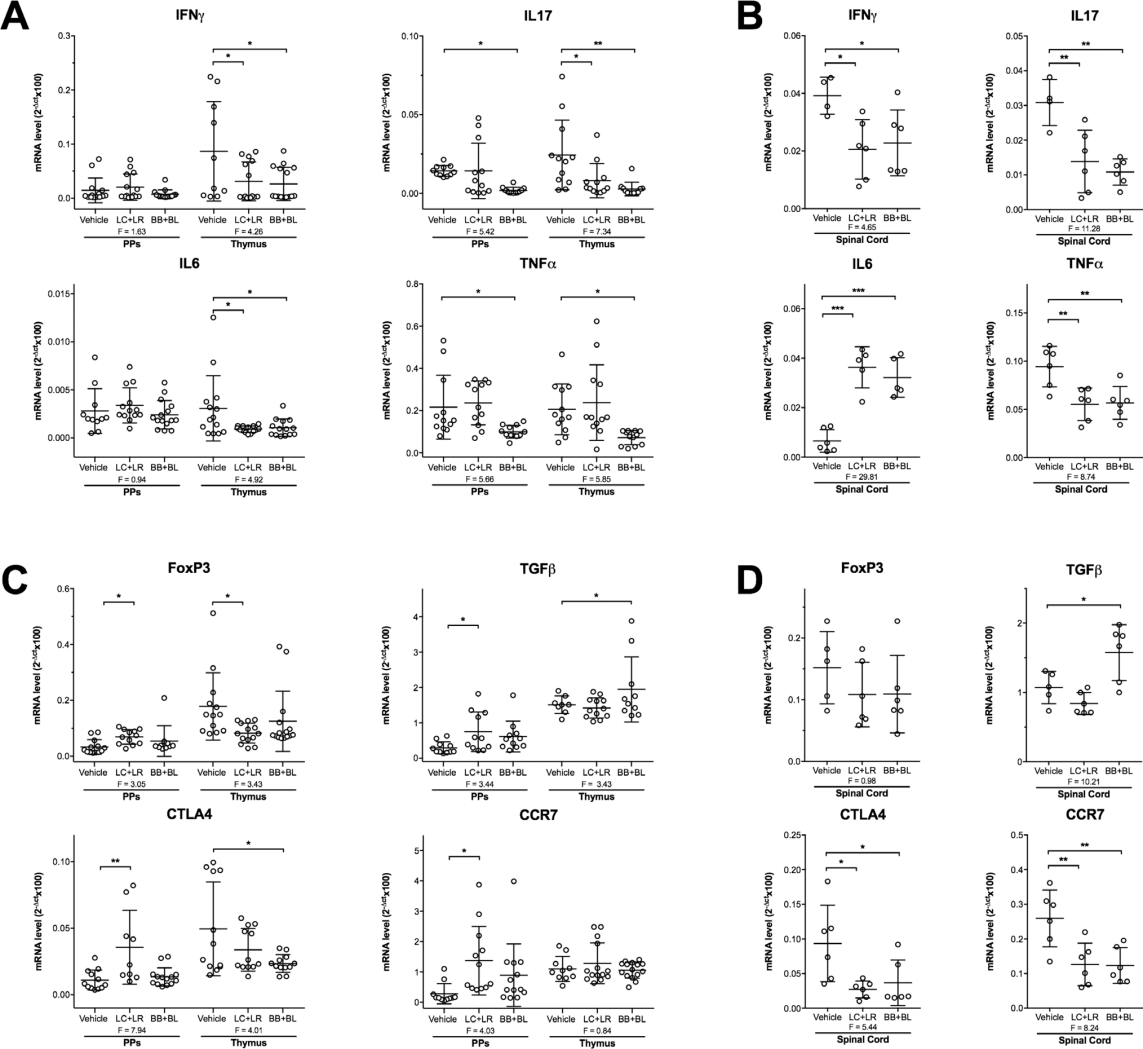
Differential expression of pro-inflammatory and immunoregulatory transcripts in EAMG (week 10 p.i.) and EAE (day 20 p.i.) rats (**A**) qRT-PCR analysis of IFNγ, IL17, IL6, and TNFα (pro-inflammatory) mRNAs in PPs and thymus of EAMG rats and (**B**) in spinal cord of EAE rats. (**C**) qRT-PCR analysis of FoxP3, TGFβ, CTLA4, and CCR7 (immunoregulatory) mRNAs in PPs and thymus of EAMG rats and (**D**) in spinal cord of EAE rats. Animals were treated with vehicle, LC+LR or BB+BL. mRNA values were normalized to β-actin as housekeeping gene and expressed as mean 2^-Δct^ × 100, *n* ≥ 8 for EAMG, *n* = 4–6 for EAE. Scatter dot plots with mean ± SD. Statistical significance was assessed by one-way ANOVA test with Dunnett's multiple comparison test; ANOVA F values are reported. ^*^P < 0.05, ^**^P < 0.01, ^***^P < 0.001.

Next, we studied whether in EAMG and in EAE animals the probiotic treatments induced modulation of IFNγ, IL17, IL6 and TNFα (pro-inflammatory markers) and FoxP3, TGFβ, CTLA4 and CCR7 (immunomodulatory markers) in PPs and thymus of EAMG rats, and in spinal cord of EAE animals, by qRT-PCR (Figure 5). Administration of LC+LR and BB+BL altered selected mRNA transcripts in PPs. Among molecular targets investigated, IFNγ, IL17, IL6 and TNFα were significantly down-regulated in EAMG thymuses (Figure 5A). IFNγ, IL17, and TNFα were also down-regulated in EAE spinal cord while IL-6 was found significantly up-regulated (Figure 5B). Differential expression of immunoregulatory FoxP3, CTLA4 and TGFβ transcripts in EAMG thymus (Figure 5C), and of TGFβ, CTLA4, and CCR7 in EAE spinal cord (Figure 5D) was observed.

### Probiotics induce immunomodulatory phenotype in DCs and release of TGFβ

**Figure 6:**
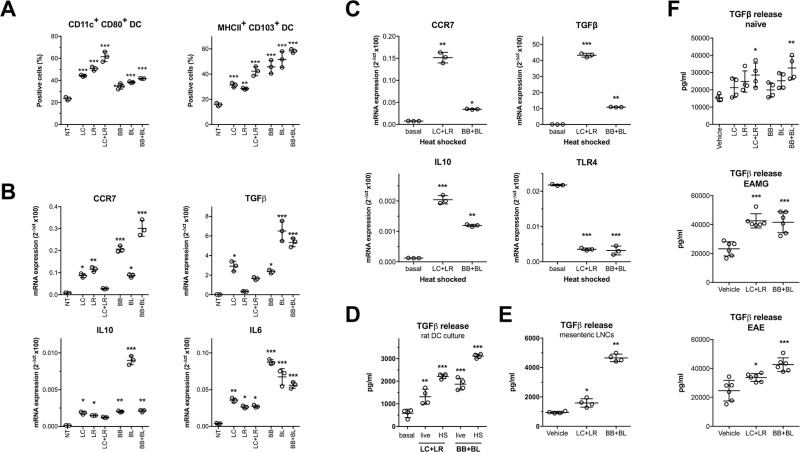
*In vitro* and *ex vivo* immunomodulatory effects of probiotics (**A**) Cytofluorimetric analysis of CD11c^+^/CD80^+^ and MHCII^+^/CD103^+^ DC incubated with single probiotic strains and combinations. (**B**) qRT-PCR analysis of CCR7, TGFβ, IL10 and IL6 immunomodulatory mRNAs in rat DCs treated with single probiotic strains and combinations. (**C**) qRT-PCR analysis of CCR7, TGFβ, IL10 and TLR4 mRNAs in DCs treated with heat shocked (HS) probiotic combinations. (**D**) TGFβ ELISA assay in supernatants of DCs treated with live or heat-inactivated (HS) probiotic combinations. (**E**) TGFβ ELISA assay in supernatants of mesenteric lymph node single cell suspensions isolated from rats treated with vehicle, LC+LR, and BB+BL (10 doses). (**F**) TGFβ ELISA assay in sera of naïve rats (top) treated with vehicle, LC, LR, LC+LR or BB, BL, BB+BL (10 doses) and EAMG rats (middle) and EAE rats (bottom) treated with vehicle and probiotic combinations (LC+LR and BB+BL). Scatter dot plots with mean ± SD. Statistical significance was assessed by one-way ANOVA test with Dunnett's multiple comparison test. ^*^P < 0.05; ^**^P < 0.01; ^***^P < 0.001.

It has been suggested that probiotics modulate immune responses by influencing dendritic cells maturation and by driving bone-marrow derived DCs to develop Treg cells [18]. Hence, we investigated whether the single probiotic strains or LC+LR and BB+BL combinations were capable to induce a immunomodulatory profile in *in vitro* cultured DCs from naïve rats. Flow cytometric analysis demonstrated DCs maturation, by increased percentages of CD11c^+^/CD80^+^ (LC+LR 62%; BB+BL 41%) and MHCII^+^/CD103^+^ (LC+LR 42%; BB+BL 58%) double positive cells (Figure 6A). Probiotic-induced DCs maturation was accompanied by increased expression of the immunomodulatory CCR7, TGFβ, IL10, and IL6 mRNAs compared to not treated (NT) DCs, more striking when the cells were exposed to bifidobacteria strains (Figure 6B). Increased CCR7, TGFβ, IL10 mRNAs was seen when DCs were exposed to heat-shocked (HS) probiotic strains; TLR4 mRNA expression was reduced (Figure 6C).

Mature DCs, induced by probiotics, retain the ability to drive Treg differentiation and, in this process, TGFβ is a key molecular mediator. Hence, we focused on the upregulated TGFβ levels found in response to our probiotic treatment; by ELISA assay, increased level of TGFβ protein was observed in the culture supernatants of DCs treated with live and HS LC+LR or BB+BL (Figure 6D), and in culture media of mesenteric LNs derived from animals fed with probiotic combinations (Figure 6E). Moreover, increased TGFβ protein levels were consistently observed in the sera of naïve rats fed either with single strains or with probiotic combinations, and in the sera of both EAMG and EAE animals fed with LC+LR or BB+BL combinations (Figure 6F).

## DISCUSSION

First-line therapies for neurological autoimmune diseases relies on generalized immunosuppression that, being not antigen-specific, often is associated with side effects due to the chronic use; hence the search for new and possibly more targeted therapies may help to improve quality of life in patients. The microbiome plays a crucial role in shaping the peripheral immune homeostasis and in the control of host susceptibility to autoimmune diseases [1, 40]. By interacting with the microbiome, probiotic bacteria provides health benefits by different mechanisms, such as regulating gut microbial homeostasis (eubiosis), restoring gastrointestinal barrier, and balancing local and systemic inflammatory immune responses, with a minimized risk of side effects [18, 41, 42]. Efficacy of probiotic treatments have been successfully investigated in several experimental models of autoimmune diseases, such as type 1 diabetes [43], rheumatoid arthritis [44], EAMG [45], and EAE [46, 47].

In the rat EAMG model, Chae *et al*. used a mixture of five probiotics (one streptococcus, one bifidobacterium and three lactobacillus strains) and demonstrated the prophylactic efficacy on EAMG [45]; animals were treated starting 2 weeks before TAChR immunization, receiving 40 consecutive probiotic administrations (10^10^ CFU/rat). Differently, we focused our study on two lactobacillus (LC and LR) and two bifidobacterium (BB and BL), selected from a larger panel on the basis of their ability to grow, adhesion to human mucus, and production of conjugated linoleic acid (CLA), an anti-oxidant and anti-inflammatory molecule (M. Elli, personal communication) [21, 39]. The probiotic strains have been individually evaluated in preliminary experiments and subsequently in combination (LC+LR and BB+BL), looking for genus specific immunomodulatory capabilities in modulating disease manifestation in EAMG and EAE rat models.

Probiotics were given to naïve healthy animals as single strains (not shown) or in combination (LC+LR and BB+BL, Supplementary Figure 1), and showed genus-specific and treatment-related immunomodulatory profiles: FoxP3, TGFβ, CTLA4 and CCR7 mRNAs were significantly upregulated by lactobacilli at week 2 (Supplementary Figure 1C), while bifidobacteria effects were seen at week 4 (Supplementary Figure 1D). The observed FoxP3 upregulation at mRNA level was assessed by FACS analysis, with increased percentages of FoxP3^+^CD4^+^CD25^bright+^ Tcells in PPs, mesenteric LNCs and PBL (Supplementary Figure 1E–1F), confirming that our probiotics mobilizes regulatory T cells in the GALT as well as in the periphery. This mechanism has been proposed to explain the IRT5 probiotic mixture effect on the systemic immune system in EAE models [17, 18, 46].

Then, lactobacilli and bifidobacteria were given to TAChR-immunized EAMG rats; both treatments significantly ameliorated EAMG symptoms, being BB+BL combination slightly more potent than LC+LR (Figure 1B and 1C). Clinical efficacy was confirmed by a significant reduction of the pathogenic anti-ratAChR Ab titre (Figure 1D), and by increased AChR content in the muscle (Figure 2). We suggest that this could be related to the different dynamic of bifidobacteria gut colonization compared to lactobacilli, as demonstrated by stool analysis in naïve rats (Supplementary Figure 1A), even if we cannot exclude strain-specific immunoregulatory mechanisms, as reported by other authors [18, 48, 49].

To further address immunoregulatory mechanisms associated to lactobacilli or bifidobacteria, we investigated the immunomodulatory profiles induced by probiotic administration in the GALT: following oral administration, probiotics were found in proximity to intestinal villi and PPs (Supplementary Figure 1B), and induced a significant downregulation of pro-inflammatory and upregulation of immunomodulatory mRNAs in naïve (Supplementary Figure 1C and 1D) and in EAMG (Figure 5A and 5C) PPs. Preliminary confocal microscopy experiments indicated that probiotics were found in association with DCs and CD3^+^ Tcells in PPs and in villi mucosa (not shown). However, the role of GALT microenvironment deserves further investigations to analyse in details probiotic interactions with the resident immunocompetent cells.

In human MG, the thymus has been suggested to be the main site of the autosensitisation process against the AChR, due to the presence of AChR-specific T cells, ectopic germinal centers of B cells, and autoantibody-producing plasma cells, all contributing to trigger the intra-thymic immune response to AChR [50, 51]. Therefore, we investigated whether in the EAMG model, pro-inflammatory and immunomodulatory transcripts in the thymus gland were modified by the probiotic treatment. Pro-inflammatory mRNAs were found downregulated (Figure 5A), while TGFβ was found increased among immunomodulatory mRNAs (Figure 5C).

In probiotic-treated naïve animals, FACS analysis revealed increased percentages of CD4^+^CD25^bright+^ and FoxP3^+^CD4^+^CD25^bright+^ regulatory Tcells not only in PPs and in mesenteric LNCs, but also in PBLs (Supplementary Figure 1E and 1F), in spleens and in cervical LNs (not shown). In MG patients and in the EAMG model, AChR-specific effector T cells are not efficiently balanced by the regulatory T cell compartment [29, 52, 53]; our data suggest that probiotic treatment results in the induction of peripheral regulatory Tcell in EAMG animals, similarly to what we observed in probiotic-fed naïve animals. Indeed, Treg induction has been demonstrated in mice after administration of selected combination of Clostridia strains [54], and in experimental murine colitis after treatment with specific bacterial antigens from Parabacteroides [55].

Bifidobacteria and lactobacilli were also effective in a different experimental autoimmune model, the MBP-induced EAE in Lewis rats; this model was selected because it has been reported to be useful to evaluate the effects of innovative therapeutic approaches modulating effector Tcell activation [56, 57], and inducing regulatory T lymphocytes; both these events are associated with EAE remission [37, 58].

In the MBP-EAE model, probiotic treatments led to significant clinical amelioration (Figure 3B), associated with preserved myelin content (Figure 3D and 3E), reduced astrocytosis, and reduced CD3^+^ Tcell infiltration (Figure 3F and 3G) in the spinal cord. We have investigated astrocyte activation, because these cells respond to CNS insults through a process referred to as reactive astrogliosis (revealed by GFAP staining), which become a pathological hallmark of CNS lesions. Astrocytes are key players in driving CNS inflammation and are directly implicated in the pathophysiology of EAE [59, 60].

To our knowledge, efficacy of a mixture of probiotics was only demonstrated in the murine EAE model induced by myelin oligodendrocyte glycoprotein peptide 35–55 [46, 47]. In a different EAE model, induced in Lewis rats with a homogenate of guinea pig spinal cord, two probiotic bacterial strains, *Lactobacillus casei* strain Shirota and *Bifidobacterium breve* strain Yakult, were evaluated but without any significant effect on disease manifestations [61].

The fermentation of dietary carbohydrates in the gut by bacteria produces short chain fatty acids (SCFAs), saturated aliphatic organic acids that consist of one to six carbons of which acetate (C2), propionate (C3), and butyrate (C4) are the most abundant [62]; SCFAs represent signaling molecules between the gut microbiota and the host [63]. In a rat model of transient focal cerebral ischemia, butyrate attenuated blood-brain-barrier (BBB) disruption [64]; moreover, oral treatment with butyrate of germ-free mice induced increased expression of occludins, key tight junction proteins in cerebral endothelial cells with an important role in modulating BBB functions and permeability [65]. In our probiotic-treated EAE rats, we demonstrated a significant reduction of CD3^+^ infiltrating T cells in the spinal cord. This observation suggested us to indirectly test BBB cell permeability by injecting in the blood stream *in vitro* expanded MBP-specific EGFP+/– Tcell blasts. Fluorescence microscopy and FACS analyses demonstrated significantly reduced numbers of EGFP+/– Tcell blasts within spinal cord in probiotic treated animals (Figure 4A and 4C); EGFP+/– Tcell blasts were found accumulated in the spleen (Figure 4B and 4D) and in PBL (Figure 4E); these results suggest that in our model the preservation of BBB cell permeability could be a consequence of probiotic treatment.

Among the investigated pro-inflammatory molecules in the spinal cord of MBP-EAE rats, all but not IL-6 were found downregulated; indeed, IL-6 mRNA level was significantly increased in treated rats (Figure 5B). In this regard, IL-6 upregulation in the CNS of EAE mice has been associated with a less aggressive disease course and with the acquisition of a protective phenotype in astrocytes in which the NF-kB dependent inflammatory pathway has been selectively inactivated [60]. Immunomodulatory mRNAs were also differentially expressed in response to probiotic treatments. TGFβ was found significantly increased by bifidobacteria treatment only, while CTLA4 (CD152) and CCR7 reduced by both bifidobacteria and lactobacilli (Figure 5D). The observed TGFβ mRNA increase in the spinal cord is similar to what was seen in EAMG thymus, thus suggesting that this molecule has indeed a critical role in the mechanisms associated to probiotic effect. Downregulation of CTLA4 and CCR7 mRNAs is also an interesting observation that deserves further investigation. Indeed, both molecules are involved in Th1 lymphocytes trafficking, as it has been reported that T cells presenting similar levels of CCR5 and CCR7 expression show improved migration following CTLA4 (CD152) signal [66]. Reduced expression of CTLA4 and CCR7 suggests that also the recruitment of effector Tcells in spinal cord has been halted, at least partially.

There are emerging evidences that anti-inflammatory effects exerted by probiotic strains are associated to the induction of regulatory T cells, although the molecular mechanism has not been elucidated yet [46, 67]. Therefore, a better understanding of the protective role of probiotics is still needed and may indicate innovative mechanisms used to induce regulatory Tcells to treat systemic autoimmunity. The possible immunomodulatory mechanisms associated with probiotic effect were investigated *in vitro* with rat bone-marrow derived DCs; our results showed that probiotic-exposed DCs were induced to differentiate towards a mature cell phenotype (Figure 6A), and this was accompanied with differential expression of CCR7, TGFβ, IL-6, and IL-10 mRNAs (Figure 6B). Similar results were obtained with inactivated (heat-shocked) probiotics (Figure 6C), suggesting that components of the bacterial wall, such as proteoglycans, could be involved in the process of DC immunomodulation, as suggested by others [39].

Among the differentially expressed transcripts, TGFβ have been further investigated by ELISA assays, and found to be increased not only in the supernatants of rat DCs exposed to probiotics (Figure 6D), but also in the supernatants of mesenteric LNCs isolated from probiotic fed rats (Figure 6E), and, more interestingly, in the sera from probiotic fed naïve as well as EAMG and EAE rats (Figure 6F). Our data demonstrate that probiotic administration skews GALT-resident DCs toward an immunomodulatory phenotype, and this is associated with increased release of TGFβ. Indeed, probiotic metabolic by-products, such as SCFAs or bacterial proteoglycans, could be seen by the immune system as regulatory signals, affecting the balance between pro- and anti-inflammatory cells by extrathymic differentiation of Treg cells [68]. Whether our bifidobacterium strains produce higher level of SCFAs compared to lactobacilli requires further investigations, addressing also the potential effects of SCFAs on Treg expansion and BBB integrity in our experimental models.

Although preclinical studies in experimental models should consider preventive as well as therapeutic protocols (i.e. in the rat EAMG model, starting with the treatment after the acute phase or alternatively, 4 weeks after immunization), prevention of disease relapses is still a relevant objective, at least in MG [28]; indeed, our results on the EAMG model demonstrated probiotic efficacy during disease manifestation (acute and chronic phases). To our knowledge, probiotic treatment in the rat EAMG was investigated by Chae *et al.*, demonstrating the efficacy of prophylactic administration (i.e. given two weeks before immunization), but no effect was seen when the probiotic mixture was given during acute stage of EAMG development [45]. With regard to the rat EAE model, probiotic treatment was evaluated but without showing any effect on disease manifestations [61]; the experimental disease was induced in Lewis rats with different antigenic preparations, i.e guinea pig spinal cord homogenate and guinea pig MBP. It will be of interest to verify the efficacy of our probiotic treatments also in these two EAE models. Clinical efficacy of probiotic combinations has been investigated in different experimental models of autoimmune diseases, in both mouse and rat, by several groups; our probiotic combinations, and in particular the two bifidobacteria strains, should be evaluated in the mouse EAMG and EAE (relapsing-remitting) models to provide further information on the mechanisms associated to the clinical effect.

Our EAMG and EAE experiments in the Lewis rat model demonstrated that probiotics induced immunomodulatory effects, although not antigen-specific. Co-administration of probiotics with immunomodulatory properties together with immunodominant epitope(s) derived from autoantigen(s) should combine immunomodulation and antigen-specificity of the treatment. On the other hand, probiotic supplementation could be associated to current immunosuppressive therapies – characterized by side effects – contributing to the systemic immunomodulation. However, despite the potential benefits of probiotic administration, concerns are raising on the safety of probiotics. This issue has been recently investigated in a meta-analysis study [69] and, although no major side effects due to probiotics have been described, it has been suggested that a safety profile for any probiotic strain/combinations should be assessed to determine the potential risks in susceptible patients, such as those treated with immunosuppressive drugs.

Our data further supports that selected probiotic strains are capable of promoting a condition of immunological tolerance, and this could be of potential therapeutic interest for the treatment of autoimmune diseases; our encouraging preclinical experiments in EAMG and EAE should rapidly translated in clinical trials assessing probiotic efficacy in human autoimmune diseases.

## MATERIALS AND METHODS

### Animals

Female Lewis rats, 6–8 weeks old, were purchased from Charles River Laboratories Italia (Calco, Italy) and housed at the animal facility of the Institute. Enhanced Green Fluorescent Protein (EGFP) transgenic Lewis rats [70] (a kind gift from Dr. Naoto Kawakami, Max Planck Institute of Neurobiology, Martinsried, Germany) were used to generate EGFP+/– MBP-specific Tcell blasts. Overall, 185 female Lewis rats were employed. Procedures involving animals were approved by the Institute Ethical Board and Italian Ministry of Health (INNCB codes: IMP-03-11, IMP-04-11, and 1064/2015-PR) and were performed in respect to the Italian Principle of Laboratory Animal Care (DDL 116/92 and DLgs 26/2014), in accordance to European Communities Council Directive 86/609/EEC and 2010/63/UE. Animals were sacrificed after deep anesthesia obtained by carbon dioxide; low-grade anesthesia with 2% isoflurane (60:40 N_2_O:O_2_, flow rate 0.8 L/min) was induced in animals prior to immunizations and treatments.

### EAMG and EAE experimental models

Experimental TAChR-EAMG [28, 71] and MBP-EAE [36] models were induced by a single subcutaneous immunization in the hind limbs (multiple sites) with 50 μg of purified TAChR (from *Torpedo Californica* electric organ; Aquatic Research Consultants) or 200 μg of MBP (from guinea pig brain; Sigma), emulsified in Complete Freud Adjuvant (CFA; Difco, 1:1 ratio), in a total volume of 200 μl. Animals were randomized before each treatment, and study personnel were blinded to the treatment group allocation. Treatment groups consisted of 6–9 animals, unless otherwise specified in figure legends. Thymus, spleen, spinal cord, muscle, gut, mesenteric, inguinal and popliteal lymph node, blood, serum and stool samples were collected, and immediately processed pending analyses.

### TAChR preparation

TAChR was purified from *Torpedo californica* electric organ according to the alkali-stripped membrane protocol [72, 73], with minor modification. Briefly, the electric organ tissue was homogenized in 10 mM sodium phosphate buffer, 1 mM EDTA, 0.02% NaN_3_, 0.01 mM PMSF, pH 7.8 for 3 minute, high speed. The extract was centrifuged for 1 hour at 100.000 × g. Pellet was resuspended in ice-cold water and the pH adjusted to 11.0 with NaOH; the membranes were immediately centrifuged for 30 minutes at 100,000 × g. TAChR was solubilized from membranes with 2% sodium deoxycholate, overnight at 4°C, then centrifuged at 100,000 xg for 1 hour. The purified receptor was analysed on SDS-PAGE. TAChR concentration was determined as [^125^I]-αBTX binding sites/ml, and protein concentration (mg/ml) by the BCA Protein Assay Kit (Thermo Scientific). Sodium deoxycholate was partially removed by progressive dialysis (1%, and then 0.05%), and TAChR aliquots stored at –80°C.

### EAMG and EAE clinical evaluation

Each animal was weighed and scored at the beginning of each experiment, and at least twice weekly until the end of the experiment; clinical scores were taken every 24 h or less if the animals demonstrated severe weakness [28, 53]. EAMG clinical score was assessed after exercise for 30 seconds, using the grip strength test. Disease severity was graded as follows: grade 0, normal strength and no abnormalities; grade 1, mildly decreased activity and weak grip or cry; grade 2, clinical signs present before exercise (tremor, head down, hunched posture, weak grip); grade 3, severe clinical signs at rest, no grip, moribund; grade 4, sacrifice, humane endpoint. EAMG was confirmed by Prostigmine test (i.p. injection). Animals were sacrificed ten weeks post TAChR/CFA immunization. EAE clinical score [32] was assessed for the presence of neurologic signs, according to the following five-point scale: 0, healthy; 1, tail weakness or paralysis; 2, paraparesis (incomplete paralysis of one or two hind limbs/plegia of one hind limb); 3, paraplegia extending to the thoracic level; 4, forelimb weakness or paralysis with hind limbs paraparesis or paraplegia; and 5, sacrifice, humane endpoint. Randomly selected animals were sacrificed at maximum worsening (EAE disease peak, day 18–20), and at the end of experiments (day 32).

### Probiotic strains and treatment protocols

The following strains were used: *Lactobacillus crispatus* LMG P-23257 (LC), *Lactobacillus rhamnosus* ATCC 53103 (LR), *Bifidobacterium animalis* subsp. *Lactis* BB12^®^ (BB, from CHR Hansen, Denmark), *Bifidobacterium animalis* subsp. *Lactis* LMG S-28195 (BL). These strains were selected from a panel of 15 probiotics (available at AAT laboratory), on the basis of their ability to grow, adhesion to human mucus, and production of CLA, an anti-oxidant and anti-inflammatory molecule (M. Elli, personal communication) [21, 39]. All strains were grown at AAT laboratory; lactobacilli were grown in De Man, Rogosa & Sharp (MRS) broth (Difco) at 37°C in microaerophilic conditions for 18 h, bifidobacteria were cultured in MRS broth supplemented with 0.05% cysteine at 37°C by anaerobic incubation for 24–48 h. Enumeration of viable bacterial cells was performed on selective media (MRS for lactobacilli, and Transoligosaccharide propionate agar medium added with 50 μg/ml mupirocin for bifidobacteria) by decimal counts. Bacteria were resuspended as single strain aliquots (10^9^ CFU/150 μl) in PBS, 20% glucose, 10% glycerol and stored at –80°C until use. Frozen aliquots were assessed for bacterial viability, and resulted to be less than 2–4% over two months storage period. Lactobacillus (LC, LR) and bifidobacterium (BB, BL) strains, alone or in combinations (LC+LR and BB+BL) were orally administered at a cumulative dose of 2 × 10^9^ CFU/300 μl.

Colonization experiments in naïve rats were performed by oral administration of 15 probiotic doses (2 × 10^9^ CFU, 300 μl) over three weeks, and stool samples were collected at baseline (control, week 0), at week 1, 2, 3, and one week after the end of probiotic administration (wash-out, week 4); randomly selected animals were sacrificed at week 2 (10 doses), and the remaining at week 4. Spleen, gut, cervical, mesenteric, inguinal and popliteal lymph nodes, PBL and serum were collected, pending analyses.

Treatment schedule in the EAMG model: probiotic administrations were given in order to cover the acute (10 doses, after immunization) and the chronic (20 doses, from day 30) phases of the disease. Treatment schedule in the EAE model: in order to fed a minimum of 15 doses, probiotic administration started one week before MBP/CFA immunization (5 doses), and continued after disease induction (10 doses).

### Microbial analysis in stool samples

Collected stool samples were stored at –80°C pending analysis. Briefly, samples were decimally diluted into sterile saline solution and plated in parallel dishes (anaerobic conditions, 37°C, 72 h) to enumerate total lactobacilli and bifidobacteria. The mean CFU value was determined in parallel plates, derived from a decimal dilution yielding a 30–300 CFU/plate.

### Muscle AChR content

Muscle AChR content was assayed as described previously [28, 71]. Briefly, AChR was solubilized from muscle membranes overnight at 4°C in Tris-HCl (pH 7.5), NaCl, PMSF, EDTA, plus 2% Triton X-100, after a centrifugation at 17,000 × g for 1 h. Solutions containing solubilized AChR were clarified by centrifugation at 100,000 × g for 30 min. AChR crude extracts (100 μl, duplicates) were incubated with [^125^I]-αbungarotoxin (αBTX) (PerkinElmer) 4 h at room temperature, transferred on DE-81 DEAE disks (Whatman) and washed with Tris-HCl buffer 0.5% Triton X-100. Radioactivity was determined by a gamma counter (PerkinElmer). The aspecific binding was subtracted from each sample by parallel tubes pre-incubated with unlabelled αBTX (Sigma). The results were expressed as fentomoles of [^125^I]-αBTX binding sites per gram of muscle.

### Anti-rat AChR Abs titre in serum

Anti-rat AChR antibodies were assayed in individual sera by conventional radioimmunoprecipitation [74]. Briefly, AChR was extracted from rat muscle and labelled with 2 nM [^125^I]-αBTX. Sera were incubated over night with labelled rat AChR (0.5 pmol). Ab-AChR complexes were precipitated by adding an excess of rabbit anti-rat IgG (Sigma). Pellet were washed twice with cold PBS plus 0.5% Triton X-100 (Carlo Erba) and [^125^I]-αBTX labelled rat AChR was evaluated by a gamma counter (Perkin Elmer). Serum samples incubated with rat AChR pre-incubated in excess of cold αBTX (Life Technologies; aspecific binding) were subtracted from test samples. The anti-AChR Ab titres were expressed as picomole of [^125^I]-αBTX binding sites precipitated per milliliter of serum.

### EAMG muscle histological analysis

Fifteen-μm thick serial cryosections from gastrocnemius muscles (6 slices per muscle) were stained with αBTX-Tetramethylrhodamine (Thermo Fisher) to detect AChR clusters, as described by Cole *et al*. [75]; muscle was counterstained with rabbit polyclonal anti-desmin Ab, followed by Alexa Fluor-488 donkey anti-rabbit secondary Ab (Thermo Fisher); nuclei were stained with 4′,6-diamidino-2-phenylindole (DAPI) (Thermo Fisher). Maximum projection images were acquired via confocal microscopy (C1/TE2000-E microscope; Nikon) using 20x (NA 0.5) and 40x (NA 1.30) objectives and used for evaluation of AChR cluster number and size (pixel), on at least 4 adjacent image fields. Parameters for image acquisition were defined and not modified to allow comparison of fluorescence intensity as a measure of relative AChR quantification. Image analysis was performed with Image J [76] and FIJI [77] software. Structure illuminated super-resolution microscopy was used to detect detailed morphological alteration of the AChR cluster at the neuromuscular junction. Single z-scan images were acquired via a N-SIM/STORM Nikon microscope, using a 100x APO-TIRF (NA 1.49) objective, with 3D optical sectioning [78].

### EAE histological analysis

Twenty-μm thick serial cryosections from lumbar tract of spinal cord (at least 6 slices) were processed for myelin staining with Black Gold II (Histo-Chem Inc. Jefferson) as described in published papers [79, 80], and counterstained with hematoxylin and eosin (H&E, Bio-Optica); images were then digitalized (ScanScope, Aperio Technologies) [81]. Single and double immunofluorescence stainings were performed on ten-μm thick spinal cord cryosections with primary Abs specific for CD3, CD4 (both from eBioscience), β-tubulin (Covance), GFAP (Dako), MBP (Chemicon). Non-immune IgG (Sigma) staining was used as isotype control. Secondary labelling was performed with Alexa Fluor 488- or 546-conjugated goat anti-mouse and donkey anti-rabbit IgGs (Thermo-Fisher); nuclei were stained with DAPI. Maximum projection images were acquired via confocal microscopy (C1/TE2000-E microscope; Nikon) over 3-micron Z stacks, acquired via 400nm-Z stepsize, using a 40x (NA 1.30) and a 60x (NA 1.40) oil objectives, and used for evaluation of CD3, MBP, GFAP and β-tubulin MFI values. Image analysis was performed with Image J [76] and FIJI [77] softwares: colour density (for BGII), MFI and single/double-positive cells were measured on at least 3 adjacent field areas per section. The workflow for the immunofluorescence analysis is given in supplementary method.

Detection of EGFP+/– cells in the spleen and spinal cord of EAE rats i.v. injected with MBP-specific Tcell blasts was performed on 10 μm-thick serial cryosections, H&E stained, and acquired via epifluorescence microscopy (6 sections/animal; *n* = 3 rats/group) using a Nikon Eclipse TE2000-E microscope.

### Probiotic localization in the gut

A single dose of fluorescently labelled (WGA-Alexa Fluor 555; Thermo Fisher) living *Lactobacillus Rhamnosus* (LR) was given to naïve rats, and the gut tissue isolated after 30 minutes. Serial 10 μm thick cryosections of Peyer's patches were co-stained with mouse anti-vimentin mAb (Dako) and sections of villi were counterstained with mouse anti-cytokeratin mAb (Dako), followed by goat anti-mouse Ab Alexa Fluor 488 conjugated. Nuclei were stained with DAPI. Single z-scan images were captured via confocal microscopy (20x objective).

### Proliferative lymph node cells responses

Proliferative responses of single cell suspensions from draining (popliteal and inguinal) lymph nodes (LN) in healthy, untreated and probiotic-treated EAMG and EAE rats were evaluated by challenging 2 × 10^5^ cells/well with 0.25 μg/ml TAChR (for EAMG), 5 and 10 μg/ml MBP (for EAE) and Concanavalin A (ConA, 2 μg/ml, Sigma). LNCs were seeded in 96-well flat plates, 200 μl RPMI medium (Euroclone) supplemented with 10% FCS, 1% Na-pyruvate, 1% non-essential aa, 1% L-glutamine, 1% penicillin-streptomycin (Euroclone), 50 μM 2-mercaptoethanol (Sigma), plus 1% normal rat serum. After 72 h of incubation at 37°C, 5% CO_2_, the cultures were pulsed with 0.5 μCi [^3^H]-thymidine/well for 18 h, and proliferation was measured from triplicate wells on a beta counter (PerkinElmer).

### EGFP+/– MBP-specific Tcell lines

EGFP+/– transgenic Lewis rats were immunized with 200 μg of MBP in CFA. Lymph nodes were aseptically removed 10 days after immunization and processed into a single-cell suspension. Lymph node cells (LNCs) were cultured in RPMI 1640 medium, 1% Na-pyruvate, 1% nonessential amino acids, 1% L-glutamine, 1% penicillin-streptomycin (Euroclone Celbio, Milan, Italy) 50 μM 2-mercaptoethanol (Sigma-Aldrich), 2% rat serum, and stimulated with 10 μg/ml of MBP. T cell lines were maintained by restimulation with MBP every 15 days, and expanded with IL-2 every 3 to 4 days thereafter. EGFP+/– Tcell blasts for i.v. injections were stimulated with MBP (10 μg/ml) and freshly prepared EGFP-/- antigen presenting cells for 2 days. MBP-specific Tcell blasts were assessed by cytofluorimetric analysis for the expression of CD4 (APC-conjugated, eBioscience) and endogenous level of EGFP; 5 × 10^6^ MBP-Tcell blasts were i.v. injected in MBP-EAE rats at day 12–14. Animals were sacrificed 4 days later to assess Tcell blasts infiltration [82, 83].

### *In vitro* cell preparation and FACS analysis

Bone marrow derived Dendritic Cells (DCs) were obtained using the classical 10-days differentiation protocol, in the presence of GM-CSF and IL4 (both 20 ng/ml; Peprotech). DCs were then seeded in 24-well culture plates (1 × 10^5^ DCs/well) and treated with viable or heat-shocked (5 min, 90°C) single strains or probiotic combinations, for 4–8 h (1 × 10^7^ CFU/well). For FACS studies, DCs were extensively washed to remove bacteria and analysed for CD11c/CD80 and MHCII/CD103 double positivity using FITC-, PE- and PerCP-labelled mAbs (eBioscience). EGFP^+^ cells in spinal cord, spleen, and PBL from EAE rats (i.v. injected with EGFP+/– MBP-specific Tcell blasts), sacrificed at disease peak (day 18–20), were quantified by FACS analysis. Spinal cord single cell suspensions were obtained from enzymatically dissociated tissues (2.5 mg trypsin and 1 mg DNAseI for 15 minutes at 37°C; both from Sigma); spleens were processed into a single-cell suspension; PBL were isolated with a density gradient medium (Lymphoprep, Nycomed). PBL, PPs, spleen, cervical LNs and mesLNs single cell suspensions from colonization experiments were stained for CD4, CD25 Tcell markers (eBioscience), APC- and PE-conjugated, together with FoxP3 (Molecular Probes, Thermo-Fisher Scientific) Alexa 488-conjugated. Samples were acquired using MACSquant (Miltenyi) and Attune NxT (Thermo-Fisher Scientific) flow cytometers. Cells were gated for Forward Scatter (FSC) and Side Scatter (SSC) parameters (BD). Aspecific staining was determined after incubation of cells with Isotype Control IgG1/IgG2a (BD). Absolute cell counts were obtained by adding a known amount of standard beads to each sample (BD).

### RT-qPCR analyses

cDNA was synthesized from total RNA (Trizol, Thermo-Fisher) using random hexamers, and reverse transcriptase (Thermo-Fisher). Real-time quantitative PCR was performed using Assay-on Demand Gene Expression Products (Thermo-Fisher) specific for: IFNγ (Rn00594078_m1), CCR7 (Rn02758813_s1), CTLA4 (Rn01437152_m1), FoxP3 (Rn01525092_m1), TGFβ (Rn00572010_m1), IL17 (Rn01757168_m1), TNFα (Rn01525859_g1), TLR4 (Rn00569848_m1), IL6 (Rn01410330_m1), and IL10 (Rn01483988_g1). β-actin (Rn01515681_m1) and GAPDH (Rn01775763_g1) were used as housekeeping endogenous genes. Target mRNA was expressed as mean 2^-Δct^x100 value, in which ΔCt is the difference between target and housekeeping Ct. Real-time PCR reactions were performed in duplicates using an ABI Prism 7500 FAST Real-Time PCR System.

### TGFβ ELISA

TGFβ release was measured in collected biological samples from naïve, EAMG and EAE animals, as well as in the supernatants of cellular cultures with a sandwich TGFβ ELISA (eBioscience), following manufacturer's instructions. TGFβ was also evaluated in supernatants from rat DCs cultures (2 × 10^6^ cells/ml) exposed to live or heat-shocked probiotic combinations (4–8 h), and from mesLNCs (2 × 10^6^ cells/ml) isolated from Lewis rats treated with LC+LR and BB+BL (10 doses) or vehicle.

### Statistical analysis

Data distribution was tested via Kolmogorov test; normally distributed data were analysed via one-way ANOVA, followed by Dunnett's multiple comparison test; corrected *p*-values for multiple comparisons were considered. EAMG and EAE clinical scores were analysed by Kruskal-Wallis test, and *p*-value corrected for multiple tests. *P* < 0.05 was considered statistically significant. GraphPad Prism v5.0 (GraphPad Prism) was used for data elaboration and statistical analyses.

## SUPPLEMENTARY MATERIALS FIGURES


