# Supplementary material for: Administration of bifidobacterium and lactobacillus strains modulates experimental myasthenia gravis and experimental encephalomyelitis in Lewis rats
Source: Oncotarget. 2018 Apr 27;9(32):22269–87. doi: 10.18632/oncotarget.25170 (PMC5976463; doi:10.18632/oncotarget.25170)
Supplement: Supplementary file 1 [file oncotarget-09-22269-s001.pdf]

Administration of bifidobacterium and lactobacillus strains modulates experimental myasthenia gravis and experimental encephalomyelitis in Lewis rats

SUPPLEMENTARY MATERIALS

Analysis of the mean fluorescence intensity

The mean fluorescence intensity of immunostainings was analysed on binary masks applied on single fluorescence channels. Background fluorescence was evaluated in isotype control stained slices followed by secondary antibody (AlexaFluor 488 or AlexaFluor 546 labelled), and subtracted from antigen-specific primary antibody staining using the "background suppression using constant" tool, prior to binary thresholding.

In details, the following command workflow for image processing was applied as a macro to all images:

- Open image file (ome-tiff file format sequence of Z-planes over 3 µm volume)
- Apply Max Projection over Z stack
- Filter: Smooth 1x
- Split color channels (Ch1: red fluorescence - Alexa Fluor 546, for CD3 labeling; Ch2: green fluorescence - Alexa Fluor488, for MBP and GFAP labelling; Ch3: blue fluorescence - for DAPI nuclear labeling)
- Apply gray-scale LUT to all single channels
- Subtract background using constant 25px

All pre-processed images were then analyzed using the following set of commands:

- Adjust binary threshold of intensity in single channel mode
- Binary object counts over field of view (object size specifications: GFAP, between 25 and 300 µm; MBP, between 10 and 150 µm; CD3, between 8 and 15 µm)
- MultiMeasure command over all objects in the field of view
- Mean fluorescence data of all objects in channel 1 (green) was averaged per field of view and represented in the graphs reported in Figure 3E–3G.

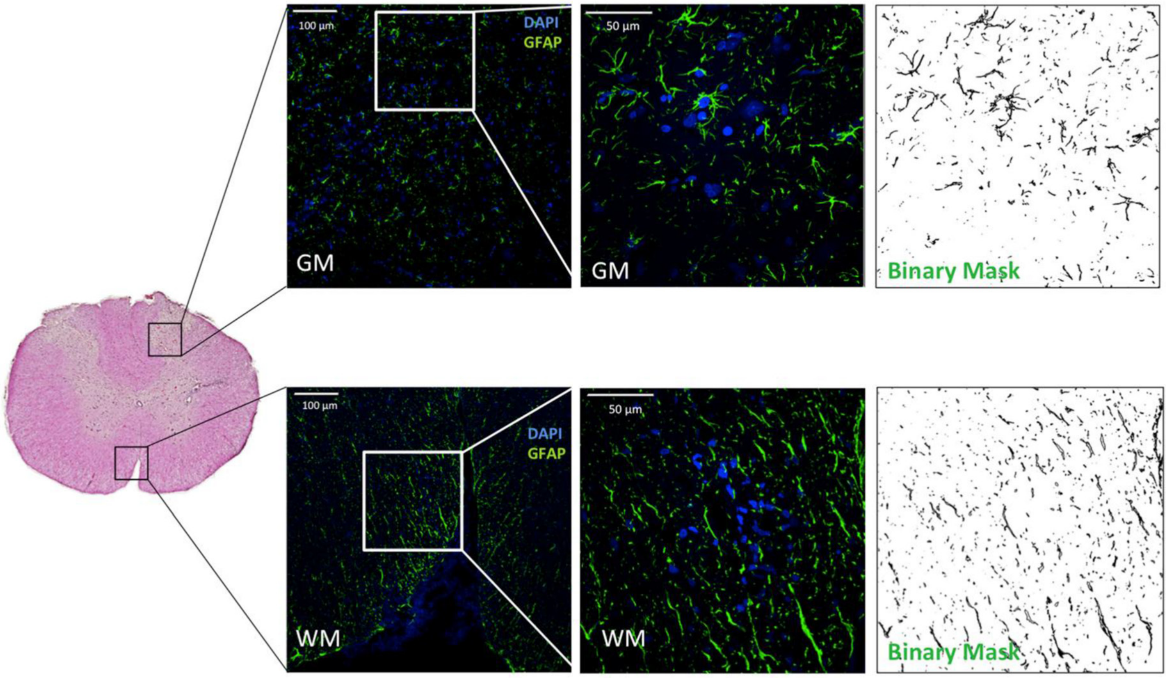

Representative EAE rat spinal cord images. E/E staining (brightfield, 2X); GFAP (AlexaFluor 488) and nuclei (DAPI) immunofluorescence (20X and 60X) microphotographs; binary masks on the green fluorescence channel used for the mean fluorescence intensity (MFI) quantification.

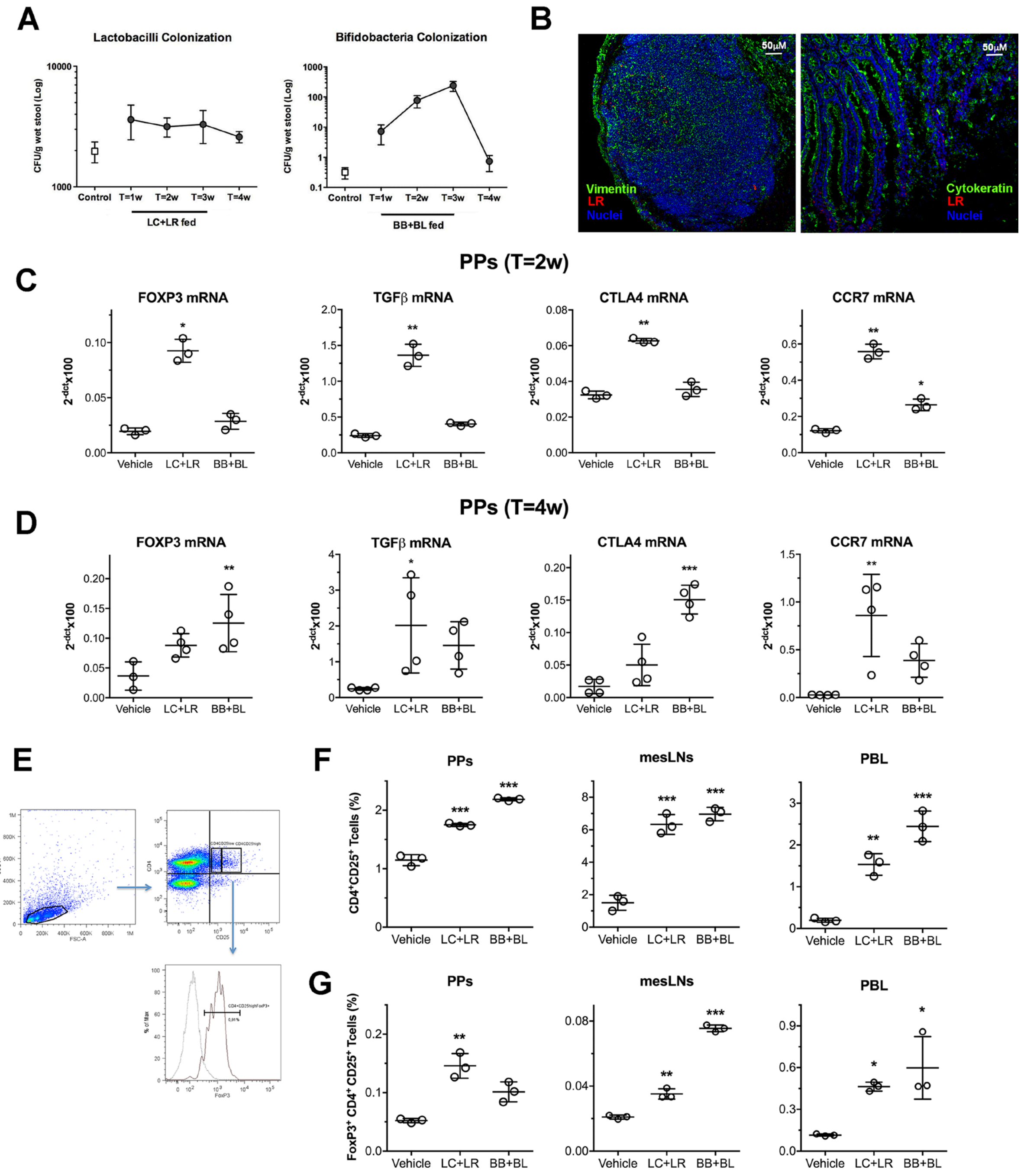

Supplementary Figure 1: Probiotics colonize the gut and induce immunomodulatory changes in PPs, mesenteric lymph nodes and in peripheral blood of naïve rats. (**A**) Total lactobacilli and bifidobacteria counts in stool samples from probiotic fed animals, at baseline, after 5 doses (T = 1w), 10 doses (T = 2w), 15 doses (T = 3w) and after one week washout (T = 4w); data are expressed as colony forming unit, CFU/g of stool samples; *n* = 3 rats/group; representative data of 2 independent experiments. (**B**) Peyer's patch and villi immunofluorescence analysis to visualize WGA-Alexa Fluor 555-labeled probiotics. PP (left panel) was counterstained for vimentin (green) and nuclei (DAPI, blue); villi (right panel) were counterstained for cytokeratin (green) and nuclei (DAPI, blue). Representative images of LR distribution. Magnification bar: 50 μm. (**C**) qRT-PCR analysis of FoxP3, TGFβ, CTLA4, and CCR7 mRNAs in PPs at T = 2w and (**D**) at T = 4w; data are expressed as $2^{-\Delta ct}$x100 normalized to β-actin as housekeeping gene and represented as scatter dot plots (mean ± SD). *N* = 6 rats/group, 2 independent experiments. (**E**) Cytofluorimetric analysis of $CD4^{+}CD25^{bright}FoxP3^{+}$ in PPs, mesenteric LNs, and PBL. Representative gating strategy with FSC/SSC, CD4/CD25 and FoxP3 histogram in $CD4^{+}CD25^{bright}$ Tcells. Percentages of (**F**) $CD4^{+}CD25^{bright}$ Tcells and (**G**) $FoxP3^{+}CD4^{+}CD25^{bright}$ Tcells in PPs, mesenteric LNs, and PBL of animals treated with vehicle, LC+LR, BB+BL, according to CD4/CD25/FoxP3 gating strategy (see Supplementary Figure 1E). Scatter dot plots with mean ± SD. Statistical significance was assessed by one-way ANOVA test with Dunnett's multiple comparison test. $^{*}P < 0.05$; $^{**}P < 0.01$; $^{***}P < 0.001$.

Clinical Score

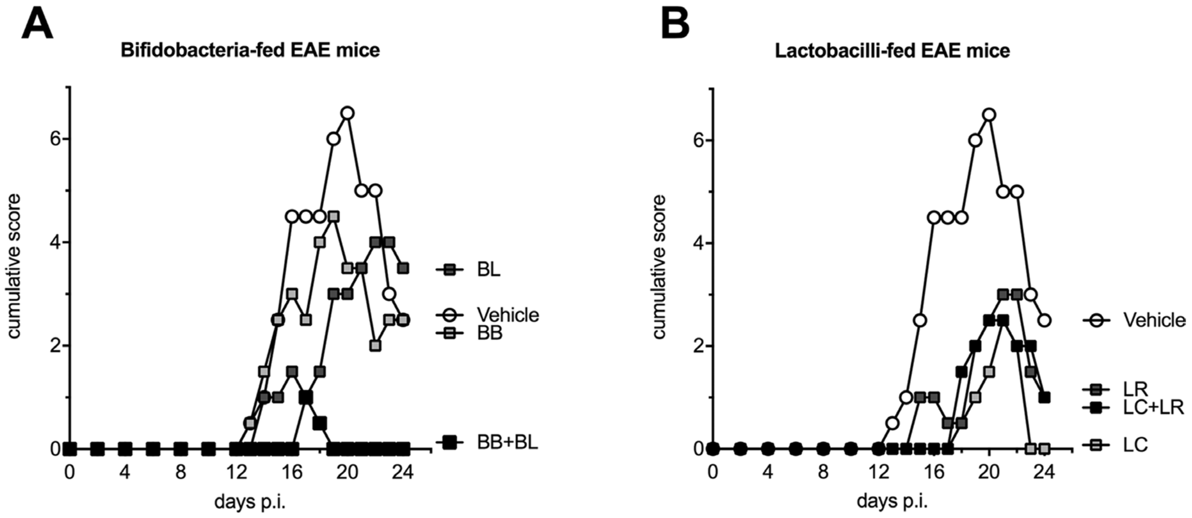

		Bifidobacteria-fed			Lactobacilli-fed		
	**vehicle**	**BB**	**BL**	**BB+BL**	**LC**	**LR**	**LC+LR**
**EAE incidence (%)**	**100**	**67**	**67**	**33**	**67**	**100**	**33**
**EAE onset (day p.i.)**	**13**	**13**	**14**	**17**	**18**	**15**	**18**
**EAE score (median, day 20)**	**2.5**	**1.5**	**1**	**0**	**0**	**1**	**0**
**total EAE score**	**40**	**27.5**	**19.5**	**1.5**	**7.5**	**13.5**	**10.5**

Supplementary Figure 2: Pilot experiment on single and combined probiotic strain administration in MBP-EAE rats. Cumulative EAE clinical score of (**A**) animals treated with vehicle, LC, LR and LC+LR and (**B**) animals treated with vehicle, BL, BB and BB+BL. Animals received 15 probiotic doses as in protocol illustrated in Figure 3A (n = 3/group). EAE incidence, EAE onset, EAE median score and total EAE score were summarized in the table.

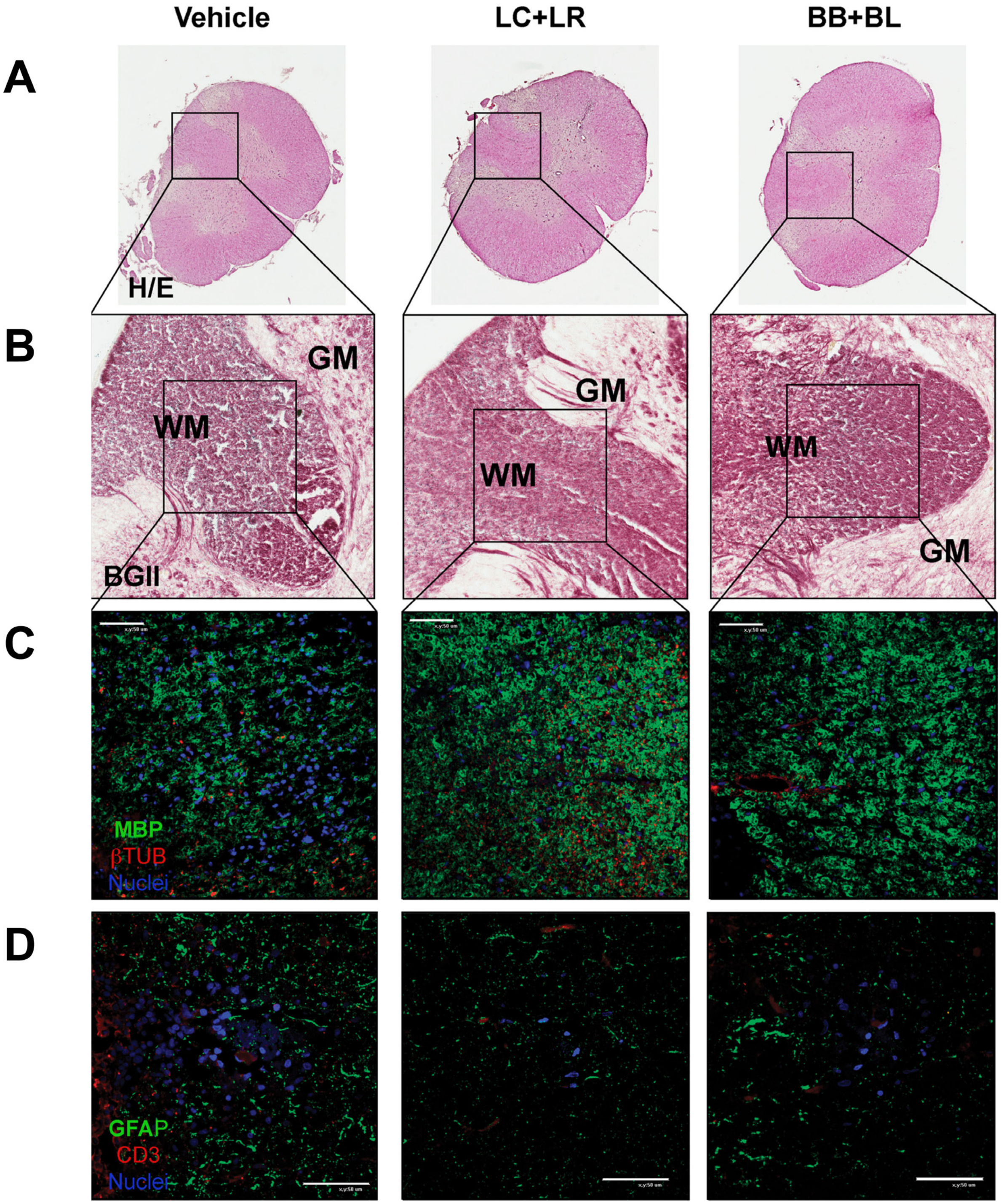

Supplementary Figure 3: Histological examination of spinal cord from EAE rats treated with probiotics. Hematoxylin/eosin staining of spinal cord (H/E; panel **A**). Histochemical staining for myelin (BGII; panel **B**); WM: white matter, GM: grey matter. Immunofluorescence staining for oligodendrocytes (anti-MBP mAb, green) and neurons (anti-β-tubulin mAb, red) (panel **C**). Immunofluorescence staining for reactive astrocytes (anti-GFAP mAb; green) and infiltrating Tcells (anti-CD3 mAb, red) (panel **D**) of thoracic spinal cord of EAE rats treated with vehicle, LC+LR or BB+BL. Nuclei were counterstained with DAPI. Scale bars: 50 µm.
